# Comparison of Surgical and Non-Surgical Treatment of Anterior Cruciate Ligament Injury in Middle-Aged Population: A Prospective Cohort Study

**DOI:** 10.7759/cureus.84489

**Published:** 2025-05-20

**Authors:** Harsh Kumar, Harjit Kanwar S Chawla, Akshay Seth, Kshitij Mehta, Girish Sahni, Mudit Sharma, Ritanshu Mangoch

**Affiliations:** 1 Orthopaedics, Government Medical College Patiala, Patiala, IND

**Keywords:** acl rehabilitation, anterior cruciate ligament (acl), arthroscopic acl reconstruction, conservative and surgical treatment, functional outcome of acl reconstruction, tegner lysholm knee scoring scale

## Abstract

Background

The anterior cruciate ligament (ACL) is one of the most commonly injured ligaments of the human body. The main mechanism of injury is usually when a pivoting injury occurs in which the tibia is translated anteriorly. At the same time, the knee is in a slight valgus and flexed attitude, and is predominantly found in contact sports like soccer. The variable symptoms that warrant medical attention usually include pain, swelling, knee giving way, difficulty ambulating, and reduced knee range of motion. The treatment usually depends on the patient's symptomatology, type of injury, the growth remaining in his or her growth plates, and activity goals. The purpose of the study is to determine the superior treatment modality for ACL injury in patients older than 40 years.

Methodology

The study was a prospective study conducted in the orthopedic outpatient department of a tertiary care center in northern India. A total of 60 patients were included in the study sample. Patients were divided into two treatment groups: Group A (n=30), consisting of conservatively managed patients, and Group B (n=30), consisting of surgically managed patients. Various symptoms during presentation were also noted in the study, which showed pain and instability as the predominant symptoms on presentation. Treatment modalities for ACL injury in our study include conservative management by physiotherapy and surgical management with ACL reconstruction using a peroneus longus autograft. The physiotherapy protocol and post-surgery rehabilitation protocol were kept the same to compare the outcome in both groups. The Lysholm knee scoring scale was used to calculate the functional outcome of conservatively and surgically managed groups before intervention and post-intervention at the first, third, sixth, and twelfth months. The data was collected and summarized in Microsoft Excel (Microsoft Corp., Redmond, WA) and analyzed with IBM Corp. Released 2012. IBM SPSS Statistics for Windows, Version 21.0. Armonk, NY: IBM Corp., using the ANOVA test, chi-square test, and paired t-test. A p-value of < 0.05 indicated a statistically significant difference.

Results

The study was conducted with two groups (group A: conservative; group B: surgical group) with comparable demographic profiles and slight male predominance, comparing the functional outcome of surgically and conservatively managed middle-aged patients with ACL injuries. The Lysholm knee scoring scale showed a poor pre-intervention score in both groups. This subsequently improved over a period of one year of study for both groups. After 12 months, a Lysholm Knee Score of 75.80±5.95 (group A) and 96.13±2.65 (group B), with a p-value of < 0.001, was noted, which showed a significantly superior functional outcome of surgical intervention in ACL injury as compared to conservative treatment in the study population.

Conclusions

Sixty patients over 40 years of age were taken up for the study, with more than two-thirds of the study population being male. The study showed the pre-intervention Lysholm knee scoring scale for both groups to be “poor.” The score remained “poor” even after the first month post-intervention. After 12 months, a Lysholm Knee Score with an “excellent” outcome was noted in the surgical group (group B), which depicted an overall superior functional outcome of surgical intervention in ACL injury as compared to conservative treatment in the study population.

## Introduction

ACL injury is one of the most common ligamentous injuries encountered in orthopedic outpatient departments [1]. The incidence of ACL injury in India is 68.6 ACL tears per 100,000 population [2]. The most common mechanism responsible for ACL injuries is road traffic accidents, followed by twisting injuries to the knee from falling [3]. Together with the other ligaments, capsule, muscles, and bone, the ACL serves as the pivot for functional congruence and stability of the knee [4]. Cruciate ligaments, which maintain the knee joint's extreme movement, are made of dense fibrous material rich in type I collagen. It is an essential component of the knee as it senses changes in the direction and location of the joint as well as variations in acceleration, speed, and stiffness [1]. ACL injuries are frequently accompanied by damage to other important structures such as the menisci, articular cartilage, and other ligaments [5]. As far as the mechanism of injury is concerned, ACL injury can occur through non-contact, indirect, or direct contact. The most frequent of the three injuries in the athletic population is the non-contact variety, which is brought on by internal forces generated by the body [6]. ACL tears are typically caused by sudden changes in direction or speed when the foot is firmly planted on the ground. Other causes include twisting, landing from a jump, pivoting, and direct contact on the front of the tibia [7]. Patients with ACL injuries typically complain of severe pain, swelling, instability in the knee joint, and hearing or sensation of a pop [1]. A crucial step in the diagnosis of an ACL tear is the physical examination. The three well-known physical tests used to assess the integrity of the anterior cruciate ligament are the pivot shift test, the anterior drawer test, and the Lachman test. Other tests, like MRIs and X-rays, are required to rule out alternative differentials, identify damage to other bony and non-bony structures, and determine the extent of ACL injury. The degree of an ACL injury may be measured by MRI, which also detects damage to other key structures such as cartilage, the menisci, other ligaments, and tendons [1]. When treating an ACL injury, the primary objective is to minimize swelling using compression, cold fomentation, and limb elevation [7]. Physical therapy or surgical reconstruction are frequently used in definitive treatment to protect long-term knee function and restore mobility [8].

ACL tears in the opposite knee are also more likely to develop after prior injury; age also poses a danger due to an increasingly active lifestyle and increased participation in sports [9].

There is still limited data that help in determining the long-term efficacy of treatment of ACL injury in patients aged 40 years and older [10]. The study aims at gathering sufficient evidence to support the appropriate treatment and guide the patients for possible expected outcomes, as very limited literature is available currently.

## Materials and methods

The study was a prospective study conducted in a tertiary care hospital in northern India. A total of 60 patients were included in the study sample. The patients were enrolled based on certain criteria: patients aged between 40+ and 65 years, no evidence of multi-ligamentous injury, no previous knee surgery, no ligamentous injury to the contralateral knee, and ACL tears confirmed radiologically with an MRI scan. Exclusion criteria used were age less than 40 years, previous ACL surgery of either knee, chronic muscle disorders, any co-existing local conditions in the form of active articular infection or inflammatory joint disease, metabolic bone disease, or neoplastic disease. Following the initial evaluation, patients with MRI-proven ACL tears who satisfied the inclusion and exclusion criteria were solicited to take part in the study. The goals of the study were thoroughly explained to each participant, who was also made aware that participation was entirely voluntary. Patients were offered both treatment options, and the pros and cons of the treatment options were explained to the patients in their vernacular language, following which the patients were divided into two treatment groups: Group A (N=30), consisting of conservatively managed patients, and Group B (N=30), consisting of surgically managed patients.

All subjects who took part in the study were explained the surgical technique for ACL reconstruction using peroneus longus tendon (PLT) autograft arthroscopically. An anteromedial portal was used to create a femoral tunnel. The anterolateral portal was primarily used for visualization. A bioabsorbable interference screw was used to secure the tibial end of the graft, and an endobutton was used to secure the femoral end of the graft. A standardized rehabilitation protocol was followed, allowing assisted weight-bearing starting on the first postoperative day. An early focus on isometric, open-chain proprioceptive exercises and quadriceps strengthening exercises was started. The patient group who chose conservative management as definitive treatment was explained the physiotherapy protocol, which was kept similar to the postoperative protocol to maintain uniformity in both comparison groups.

The Lysholm Knee Scoring scale was used to calculate the functional outcome of conservatively and surgically managed groups pre-intervention and at one, three, six, and twelve months post-intervention. Data was collected and analyzed statistically and was summarized in Microsoft Excel (Microsoft Corp., Redmond, WA) and was analyzed with IBM Corp. Released 2012. IBM SPSS Statistics for Windows, Version 21.0. Armonk, NY: IBM Corp., using the ANOVA test, chi-square test, and paired t-test. A p-value < 0.05 indicated a statistically significant difference.

Statistical analysis: A pre-structured proforma was employed across all cases to standardize data collection. The gathered information was systematically compiled and tabulated to enable the analysis of valid outcomes.

All statistical calculations were done using the IBM Corp. Released 2012. IBM SPSS Statistics for Windows, Version 21.0. Armonk, NY: IBM Corp., statistical program for Microsoft Windows.

## Results

Symptomatically, pain and instability were the most common complaints in both groups, with 18 (60%) patients of Group A (N=30) and 19 (63.33%) patients of Group B (N=30) reporting these symptoms. The presence of knee locking was more common in Group B (N=30), with six patients (20%) reporting it compared to 3 patients (10%) in Group A (N=30). Table 1 may suggest that the severity of meniscal injury in the Group B (N=30) patients might be more than that in Group A (N=30), and surgical intervention is more frequently sought in cases of mechanical symptoms. However, the difference was not statistically significant, indicating that both treatments effectively address common symptoms of ACL injuries.

**Table 1:** Symptomatology SD: Standard Deviation; p-value: Probability value Data are represented as N (%) and Mean ± Standard Deviation (SD). Statistical analysis was performed using One Way ANOVA (Analysis of Variance) test. A p-value (Probability value) of <0.05 was considered statistically significant. N = 30 patients in each group

Symptoms	Group A		Group B		p-value
	Patients	Percentage	Patients	Percentage	
Pain, Instability	18	60%	19	63.33%	0.2045 (Not Significant)
Pain	4	13.33%	4	13.33%	
Instability	4	13.33%	1	3.33%	
Pain, Instability, Locking of Knee	3	10%	6	20%	
Total	30	100%	30	100%	

In terms of the grade of ACL tear, both groups had a majority of patients with high-grade ACL tears, with 25 patients (83.33%) in both groups Table 2, which suggests that both treatment groups were comparable in severity of injury. The chi-square test (p = 1) showed no significant difference in the distribution of tear grades between the two groups, which means the severity of the tear was similar and did not influence the choice of treatment in the study subjects.

**Table 2:** Distribution of patients based on grade of tear Data are represented as N (%). Statistical analysis was performed using the Chi-Square test. A p-value (probability value) of <0.05 was considered statistically significant. N = 30 patients in each group.

Grade of tear	Group A (N=30)		Group B (N=30)		X^2^	p value
	Patients	Percentage	Patients	Percentage		
High grade	25	83.33%	25	83.33%	0	1 (Not Significant)
Low grade	5	16.67%	5	16.67%		
Total	30	100%	30	100%		

The time since injury did not differ significantly between the groups (p = 0.8075), with a mean of 68.70 ± 97.65 days for Group A (N=30) and 76.37 ± 141.15 days for Group B (N=30). While the range of time since injury was extensive, that is, from 4 to 450 days for Group A (N=30) and 5 to 730 days for Group B (N=30) (Table 3).

**Table 3:** Injury to intervention time SD: Standard Deviation; p-value: Probability value. Data are represented as N (%) and Mean ± Standard Deviation (SD). Statistical analysis was performed using Student’s t test. A p-value (Probability value) of <0.05 was considered statistically significant. N = 30 patients in each group.

Time since injury	Group A (N=30)		Group B (N=30)		t-statistic	p- Value
	Patients	Percentage	Patients	Percentage		
≤25 days	13	43.33%	14	46.67%	0.245	0.8075 (Not Significant)
26-50 days	8	26.67%	7	23.33%		
51-75 days	3	10%	2	6.67%		
76-100 days	0	0%	1	3.33%		
>100 days	6	20%	6	20%		
Total	30	100%	30	100%		
Range	4-450 days		5-730 days			
Mean± SD	68.70±97.65		76.37±141.15			

Co-morbid conditions like hypertension were more prevalent in Group B--the surgical group (N=30), with 7 patients (23.33%) having hypertension compared to 4 patients (13.33%) in Group A (N=30). Additionally, hypertension and diabetes mellitus together were more common in Group A (N=30) (Table 4). These findings suggest that patients with higher rates of systemic conditions like hypertension may be more inclined to undergo surgery, potentially due to the severity of their injury or the desire to restore knee function more effectively.

**Table 4:** Co-morbidities Data are represented as N (%). N = 30 patients in each group.

Co-morbidities	Group A (N=30)		Group B (N=30)		p-value
	Patients	Percentage	Patients	Percentage	
Hypertension	4	13.33%	7	23.33%	0.5241 (non-significant)
Hypertension, Diabetes mellitus	6	20%	3	10%	
Hypertension, Chronic Smoker, Alcoholic	3	10%	1	3.33%	
Hypertension, Diabetes mellitus, Nicotine use	0	0%	1	3.33%	
Diabetes mellitus	0	0%	1	3.33%	
No comorbidities	17	56.67%	17	56.67%	

Most patients in both groups had a full ACL tear, with 14 patients (46.67%) in Group A (N=30) and 17 patients (56.67%) in Group B (N=30). The incidence of partial ACL tears was higher in Group A (N=30) compared to Group B (N=30). The presence of concomitant meniscus tears was similar between the groups, with 11 patients (36.67%) in Group A (N=30) and 12 patients (40%) in Group B (N=30) reporting meniscal injury either with complete or partial ACL tear (Table 5).

**Table 5:** Distribution of patients according to diagnosis SD: Standard Deviation; p-value: Probability value. Data are represented as N (%) and Mean ± Standard Deviation (SD). Statistical analysis was performed using One Way ANOVA (Analysis of Variance) test. A p-value (Probability value) of <0.05 was considered statistically significant. N = 30 patients in each group

Diagnosis	Group A (N=30)		Group B (N=30)		p-value
	Patients	Percentage	Patients	Percentage	
ACL tear	14	46.67%	17	56.67%	0.6344 (Not Significant)
ACL partial tear	5	16.67%	1	3.33%	
ACL tear + meniscus tear	11	36.67%	10	33.33%	
ACL partial tear + meniscus tear	0	0%	2	6.67%	
Total	30	100%	30	100%	

Pre-intervention, the Lysholm Knee Scoring scale for both groups was poor (group A (N=30): 47.2±7.98; group B (N=30): 45.20±11.76) (Table 6).

**Table 6:** Pre-intervention score Data are represented as N (number of patients), %, Mean ± Standard Deviation (SD), and range. Grades were categorized as: Excellent (score 91–100), Good (score 84–90), Fair (score 65–83), Poor (score <65). Statistical analysis was performed using Student’s t-test. A p-value of < 0.05 was considered statistically significant. In this analysis, both Group A and Group B consisted of 30 patients each (N = 30).

Grades	Group A (N=30)		Group B (N=30)		t-statistic	p- Value
	Patients	Percentage	Patients	Percentage		
Excellent (91-100)	0	0%	0	0%	-0.771	0.4440 (Not Significant)
Good (84-90)	0	0%	0	0%		
Fair (65-83)	0	0%	3	10%		
Poor (<65)	30	100%	27	90%		
Total	30	100%	30	100%		
Range	34-64		32-68			
Mean± SD	47.2±7.98		45.20±11.76			

One month post-intervention, the mean score was found to be 60.43±6.75 and 83.67±3.72 (p<0.001), and was 72.00±5.92 and 92.20±3.87, respectively, for both groups (p<0.001) after three months.

After six months, Group B patients achieved a mean score of 95.27 ± 3.24, with 90% of patients (27/30 patients) in the "excellent" category. Group A (N=30), however, showed only modest improvement (mean = 72.80 ± 5.69), with 90% (27/30 patients) still in the "fair" category.

After 12 months, a Lysholm Knee Score of 75.80±5.95 (group A) and 96.13±2.65 (group B), with a p-value of < 0.001, was noted (Table 7). Though the Lysholm Knee Score was improving for both groups (Table 8), a significant improvement in Group B (surgical) implied a superior functional outcome of surgical intervention in ACL injury as compared to conservative treatment in the study population.

**Table 7:** Pre intervention and post intervention Lysholm Knee Score Data are represented as N (number of patients), Mean ± Standard Deviation (SD), and score range. Each group consisted of 30 patients (N = 30). Lysholm Knee Score grades were classified as follows: Excellent: 91–100; Good: 84–90; Fair: 65–83; Poor: <65. Scores were assessed pre-operatively and at 1, 3, 6, and 12 months post-intervention. Group A received conservative treatment, and Group B underwent surgical intervention. Statistical analysis was performed to compare intra-group and inter-group differences across time points using Student’s t-test. A p-value < 0.05 was considered statistically significant. A p-value of < 0.05 was considered statistically significant. A p-value of < 0.001 was considered statistically highly significant. Improvements in Lysholm Knee Score were significantly higher in Group B at all post-intervention time points (p < 0.00001).

Pre-intervention				Post-intervention Month 1			Post-intervention Month 3			Post-intervention Month 6			Post-intervention Month 12		
Grades	Group A (N=30)	Group B (N=30)	p- Value	Group A (N=30)	Group B (N=30)	p- Value	Group A (N=30)	Group B (N=30)	p- Value	Group A (N=30)	Group B (N=30)	p- Value	Group A (N=30)	Group B (N=30)	p- Value
	Patients	Patients		Patients	Patients		Patients	Patients		Patients	Patients		Patients	Patients	
Excellent (91-100)	0	0	0.4440 (Not Significant)	0	0	< 0.00001 (Highly Significant)	0	15	< 0.00001 (Highly Significant)	0	27	< 0.00001 (Highly Significant)	0	30	< 0.00001 (Highly Significant)
Good (84-90)	0	0		0	19		0	15		0	3		3	0	
Fair (65-83)	0	3		8	11		26	0		27	0		26	0	
Poor (<65)	30	27		22	0		4	0		3	0		1	0	
Total	30	30		30	30		30	30		30	30		30	30	
Range	34-64	32-68		45-72	75-89		62-82	86-99		62-82	90-100		62-86	91-100	
Mean± SD	47.2±7.98	45.20±11.76		60.43±6.75	83.67±3.72		72.00±5.92	92.20±3.87		72.80±5.69	95.27±3.24		75.80±5.95	96.13±2.65	

**Table 8:** Intra-group comparison of Lysholm Knee Score Data are expressed as Mean ± Standard Deviation (SD). Each group included 30 patients (N = 30). The Lysholm Knee Score was recorded at five timepoints: pre-intervention, and at 1, 3, 6, and 12 months post-intervention. Group A received conservative (non-surgical) management, and Group B underwent surgical treatment. A statistically significant improvement was observed in both Group A and Group B across all post-intervention timepoints using One Way ANOVA (Analysis of Variance) test. A p-value of < 0.05 was considered statistically significant; p < 0.001 indicates highly significant (HS) difference.

Timepoints	Mean Lysholm Knee Score^*^			
	Group A (N=30)	p- Value	Group B (N=30)	p- Value
Pre-intervention	47.2±7.98	< 0.00001 (Highly Significant)	45.20±11.76	< 0.00001 (Highly Significant)
Post-intervention Month 1	60.43±6.75		83.67±3.72	
Post-intervention Month 3	72.00±5.92		92.20±3.87	
Post-intervention Month 6	72.80±5.69		95.27±3.24	
Post-intervention Month 12	75.80±5.95		96.13±2.65	

## Discussion

This study aimed to compare the outcomes of conservative versus surgical treatment in patients over 40 years of age with anterior cruciate ligament (ACL) injuries. Group A (conservative treatment) and Group B (surgical treatment) were evaluated in terms of age, gender, symptomatology, time since injury, diagnosis, grade of ACL tear, co-morbidities, and functional outcomes measured by the Lysholm Knee Score. The age distribution in both groups revealed that the majority of patients were in the 41-50 years age group, with 90% (27 patients) in Group A (N=30) and 80% (24 patients) in Group B (N=30) lying in the range, with mean ages of 45.07 ± 4.86 years for Group A (N=30) and 46.13 ± 6.02 years for Group B (N=30). These findings align with previous studies that report ACL injuries predominantly affecting younger to middle-aged adults, but also a noticeable proportion of older adults (greater than 40 years). [11]. Brown CA et al. did a study on 627 patients and found the mean age to be 49 years (range, 42.6-60.0 years) [12]. Similarly, Heier KA et al. found similar results in their study, showing the average age to be 44.6 years [13]. These findings were found to be in corroboration with Corona K et al. [3], Schneider KN et al. [2], Khan RM et al. [5], and Barber FA et al. [6]. The t-test comparison (p = 0.456) indicated no significant difference in age distribution between the two groups in our study (significant p-value of < 0.05), reinforcing that age is not a distinguishing factor in treatment assignment or outcomes in this cohort. In terms of gender, both groups were predominantly male, with 23 male patients (76.67%) in Group A (N=30) and 25 male patients (83.33%) in Group B (N=30), which is consistent with general trends in ACL injuries, where men are more likely to experience ACL tears [6,12,10,14]. Similar results were found in studies done using the chi-square test (p = 0.5186), which showed no significant difference between the groups, suggesting that gender did not influence the selection of treatment or outcomes. Regarding the diagnosis, most patients in both groups had a full ACL tear, with 14 patients (46.67%) in Group A (N=30) and 17 patients (56.67%) in Group B (N=30). The incidence of partial ACL tears was higher in Group A (N=30), with 5 patients (16.67%) having partial ACL tears compared to 10 patients (3.33%) in Group B (N=30). The presence of concomitant meniscus tears was similar between the groups, with 11 patients (36.67%) in Group A (N=30) and 12 patients (40%) in Group B (N=30) having meniscal injuries (Table 3). Similar findings were found by Buss DD et al. in their study [15]. These findings suggest that both conservative and surgical treatments were applied across similar diagnoses, which is in line with prior research [16]. The time since injury did not differ significantly between the groups (p = 0.8075), with a mean of 68.70 ± 97.65 days for Group A (N=30) and 76.37 ± 141.15 days for Group B (N=30). While the range of time since injury was extensive (from 4 to 450 days for Group A and 5 to 730 days for Group B), the lack of a significant p-value suggests that the duration between injury and treatment did not influence the choice of surgical or conservative management (significant p-value < 0.05), aligning with previous studies that suggest the timing of ACL treatment does not always correlate with outcomes [17]. The Lysholm Knee Score is one of the widely accepted measures of knee functionality post-injury. Pre-intervention, both groups had similar low Lysholm scores, with most patients scoring in the "poor" category (<65), reflecting the functional limitations associated with ACL injuries. This finding is consistent with the literature, which indicates that ACL injuries significantly impair knee function, regardless of the treatment approach [18].

Brown CA et al. also found similar results where the preoperative mean Lysholm score was found to be 54.8, which on further follow-up postoperatively was found to be 91.1 [12]. Barber A et al. found Lysholm scores over subsequent follow-up to be excellent (95-100) or good (84-94) in the majority of subjects [6]. By one-month post-treatment, Group B (N=30) had a marked improvement (mean = 83.67 ± 3.72), with 19 patients (63.33%) achieving "good" scores, while Group A's scores were lower (mean = 60.43 ± 6.75), with 22 patients (73.33%) still in the "poor" category. Tsoukas et al. [19] reported a mean International Knee Documentation Committee (IKDC) score of 86.7 in the surgical group versus 77.5 in the conservative group, indicating superior early outcomes with surgery. Kessler et al. [20] (N = 60) found that 53% of patients in the surgical group achieved a normal IKDC score, compared to 14% in the conservative group, highlighting better early functional results with surgical intervention. Sandberg et al. [21] observed a Lysholm Knee Score of 90 in the surgical group, suggesting a trend toward better early outcomes. The difference between the groups in our study was statistically significant (p < 0.00001), highlighting the superiority of surgical intervention for functional recovery within the first month after treatment. At three months post-treatment, Group B (N=30) showed continued improvement (mean = 92.20 ± 3.87), with half of the patients achieving "excellent" scores and the other half achieving "good" scores. In contrast, Group A's scores (mean = 72.00 ± 5.92) were significantly lower, with 26 patients (86.67%) in the "fair" category. The difference was highly significant (p < 0.00001), indicating that surgery provides faster and better functional recovery. Seitz et al. [22] reported that both groups had similar functional outcomes, with the surgical group showing a slight advantage in knee stability. Meuffels et al. [23] found that surgical treatment resulted in better functional outcomes and higher activity levels compared to conservative management. By six months, Group B patients had achieved a mean score of 95.27 ± 3.24, with 27 patients (90%) in the "excellent" category. Group A (N=30), however, showed only modest improvement (mean = 72.80 ± 5.69), with 27 patients (90%) still in the "fair" category. Van Yperen et al. [24] and Marx et al. [25] observed that surgical patients had better functional outcomes and less knee instability at mid-term follow-up. After 12 months of follow-up, a "fair" result with a mean value of 75.80±5.95 for group A (N=30) and an “excellent” outcome with a mean value of 96.13±2.65 were found in our study. Frobell et al. [18], van Meuffels et al. [23], and Yperen et al. [24] demonstrated that surgical patients had higher activity levels and better knee function compared to those treated conservatively. The p-value of <0.00001 again reflected the significant difference in outcomes between the two groups, with surgery yielding substantially better functional recovery over time statistically. Table 9 shows a comparative analysis of the relevant literature available on middle-aged ACL injury patients.

**Table 9:** Comparative review of literature This table summarizes key comparative findings from landmark studies evaluating surgical vs. conservative management of anterior cruciate ligament (ACL) tears. Data are represented as Mean Age (in years), %, N (sample size), and qualitative outcome comparisons. Where available, sample size (N) is mentioned alongside each study. Outcomes are categorized at 1, 3, 6, and 12 months post-intervention and include patient-reported outcome measures such as the Tegner Lysholm Knee Score and International Knee Documentation Committee (IKDC) Score. A p-value of < 0.05 was considered statistically significant in referenced studies where statistical comparisons were performed. All outcome summaries reflect findings reported by the original study authors.

Study	Mean Age	Sex Predominance	Grade of Tear	Outcome at 1 Month	Outcome at 3 Months	Outcome at 6 Months	Outcome at 12 Months
Frobell et al. (2010)[18] (N = 121)	31.8 yrs	Balanced	Partial and complete	Comparable	Comparable	Slight edge to surgical	No significant difference, but fewer secondary injuries in surgical
Kessler et al. (2008)[20] (N = 60)	41.2 yrs	Male dominant (56%)	Complete	Surgical group: higher Lysholm & IKDC, better stability	Surgical superior	Continued improvement in surgery group	53% surgical patients achieved normal IKDC vs 14% in conservative
Sandberg et al. (1987)[21]	36.5 yrs	Male predominant	Complete	Surgical group higher Lysholm (~90) vs non-op (~77)	Better outcomes in surgical	Surgical showed better return to function	Conservative group showed delayed recovery
Ehlinger et al. (2021)[22]	52.3 yrs	Male (58%)	Complete	Not reported	Surgical group showed improved Lachman and pivot-shift tests	Surgical group superior in KT-1000 measurements	Improved subjective scores, fewer subsequent operations in surgical group
Meuffels et al. (2009)[23] (N=50)	40.3 yrs	Male (70%)	Complete	Not available	Better functional outcome in surgery	Surgical patients more active	Long-term surgical superiority in high-demand patients
van Yperen et al. (2018) [24] (N=50)	41 yrs	Male (60%)	Complete	Not reported	Not reported	Better function in surgical	Significantly fewer secondary injuries, better sports return in surgical
Marx et al. (2003) [25]	42 yrs	Not specified	All types	Survey-based data only	—	—	Surgical preferred by most orthopedists for high-grade tears

Limitations

Despite the significant findings of this study, several limitations must be acknowledged. The study included a relatively small sample size from a single-center study, which may not be representative of wider geographic or demographic variations, thereby restricting applicability to other healthcare settings. There might be some selection bias due to the use of simple randomization. It is important to note that surgical superiority may be influenced by unmeasured confounders (e.g., baseline activity levels, patient preferences). Also, comorbidities may have influenced treatment choice or outcomes. The follow-up period was limited to 12 months, providing only short- to mid-term outcome data, without insights into long-term consequences such as osteoarthritis or recurrent injuries. The lack of blinding in outcome assessment may also have led to observer bias, since both patients and physicians were aware of the treatment modality. While meniscal injuries were recorded, details regarding the severity, specific location, and management of these injuries were not analyzed, which may have influenced outcomes independently of ACL treatment.

## Conclusions

This study confirms that surgical treatment for ACL injuries in patients over 40 years old leads to significantly better functional outcomes as compared to conservative management. The Lysholm Knee Scores demonstrated a marked improvement in the surgical group starting from the first month and continuing through 12 months post-intervention, whereas the conservative treatment group showed limited recovery. Given the increasing prevalence of ACL injuries in older adults, these findings support the recommendation for surgical intervention in cases of ACL injury in patients over 40, especially those seeking quicker and more substantial functional recovery.

ACL deficiency can further lead to chronic pain, damage to the menisci, and injury to articular cartilage, resulting in early osteoarthritis of the knee. Thus, the ACL is protective for both menisci and articular cartilage, and hence ACL injury warrants intervention, as supported by the evidence generated in our study. Although we strongly suggest the need for further quality studies, there is a need to gather the evidence to devise a management protocol for ACL injury in the middle-aged population.
